# Finer-Scale Phylosymbiosis: Insights from Insect Viromes

**DOI:** 10.1128/mSystems.00131-18

**Published:** 2018-12-18

**Authors:** Brittany A. Leigh, Sarah R. Bordenstein, Andrew W. Brooks, Aram Mikaelyan, Seth R. Bordenstein

**Affiliations:** a Department of Biological Sciences, Vanderbilt University, Nashville, Tennessee, USA; b Vanderbilt Genetics Institute, Vanderbilt University Medical Center, Nashville, Tennessee, USA; c Department of Pathology, Microbiology and Immunology, Vanderbilt University Medical Center, Nashville, Tennessee, USA; d Vanderbilt Institute for Infection, Immunology and Inflammation, Vanderbilt University Medical Center, Nashville, Tennessee, USA; Cornell University

**Keywords:** *Nasonia*, *Proteus*, *Providencia*, *Morganella*, bacteriophage, microbiome, microbiota, phage, phylosymbiosis, virome

## Abstract

Phylosymbiosis was recently proposed to describe the eco-evolutionary pattern whereby the ecological relatedness (e.g., beta diversity relationships) of host-associated microbial communities parallels the phylogeny of the host species. Representing the most abundant biological entities on the planet and common members of the animal-associated microbiome, viruses can be influential members of host-associated microbial communities that may recapitulate, reinforce, or ablate phylosymbiosis. Here we sequence the metagenomes of purified viral communities from three different parasitic wasp *Nasonia* species, one cytonuclear introgression line of *Nasonia*, and the flour moth outgroup Ephestia kuehniella. Results demonstrate complete phylosymbiosis between the viral metagenome and insect phylogeny. Across all *Nasonia* contigs, 69% of the genes in the viral metagenomes are either new to the databases or uncharacterized, yet over 99% of the contigs have at least one gene with similarity to a known sequence. The core *Nasonia* virome spans 21% of the total contigs, and the majority of that core is likely derived from induced prophages residing in the genomes of common *Nasonia*-associated bacterial genera: *Proteus*, *Providencia*, and *Morganella*. We also assemble the first complete viral particle genomes from *Nasonia*-associated gut bacteria. Taken together, results reveal the first complete evidence for phylosymbiosis in viral metagenomes, new genome sequences of viral particles from *Nasonia*-associated gut bacteria, and a large set of novel or uncharacterized genes in the *Nasonia* virome. This work suggests that phylosymbiosis at the host-microbiome level will likely extend to the host-virome level in other systems as well.

**IMPORTANCE** Viruses are the most abundant biological entity on the planet and interact with microbial communities with which they associate. The virome of animals is often dominated by bacterial viruses, known as bacteriophages or phages, which can (re)structure bacterial communities potentially vital to the animal host. Beta diversity relationships of animal-associated bacterial communities in laboratory and wild populations frequently parallel animal phylogenetic relationships, a pattern termed phylosymbiosis. However, little is known about whether viral communities also exhibit this eco-evolutionary pattern. Metagenomics of purified viruses from recently diverged species of *Nasonia* parasitoid wasps reared in the lab indicates for the first time that the community relationships of the virome can also exhibit complete phylosymbiosis. Therefore, viruses, particularly bacteriophages here, may also be influenced by animal evolutionary changes either directly or indirectly through the tripartite interactions among hosts, bacteria, and phage communities. Moreover, we report several new bacteriophage genomes from the common gut bacteria in *Nasonia*.

## Abstract (precis)

Viruses are the most abundant biological entity on the planet and interact with microbial communities with which they associate. The virome of animals is often dominated by bacterial viruses, known as bacteriophages or phages, which can (re)structure bacterial communities potentially vital to the animal host. Beta diversity relationships of animal-associated bacterial communities in laboratory and wild populations frequently parallel animal phylogenetic relationships, a pattern termed phylosymbiosis. However, little is known about whether viral communities also exhibit this eco-evolutionary pattern. Metagenomics of purified viruses from recently diverged species of *Nasonia* parasitoid wasps reared in the lab indicates for the first time that the community relationships of the virome can also exhibit complete phylosymbiosis. Therefore, viruses, particularly bacteriophages here, may also be influenced by animal evolutionary changes either directly or indirectly through the tripartite interactions among hosts, bacteria, and phage communities. Moreover, we report several new bacteriophage genomes from the common gut bacteria in *Nasonia*.

## INTRODUCTION

Ecological similarity of host-associated microbial communities between species can often mirror phylogenetic similarity of hosts across a wide range of animal taxa (1–6). This eco-evolutionary pattern, termed phylosymbiosis (1, 7), can arise from a variety of biotic or abiotic factors. Resultantly, phylosymbiosis does not *a priori* presume stable or long-term, transgenerational associations between microbial communities and their hosts. Phylosymbiosis may change with environments, lifestyles, or multipartite interactions that shift assembly of microbial communities. For example, phages (i.e., bacteriophages; viruses that infect bacteria) can outnumber bacteria in both free-living and host-associated communities (8, 9), represent the majority of viruses within animal microbiomes (8, 10–13), and may drive or ablate bacterial phylosymbiosis as they prey on bacteria.

A phage can exhibit two main life cycles: lytic and temperate. A lytic phage infects its bacterial host and immediately replicates and lyses the bacterial cell. A temperate phage, however, can integrate into and replicate as part of the bacterial genome until a biotic or abiotic trigger causes it to excise and enter the lytic cycle. In mammalian host-associated phage communities, the temperate life cycle dominates (10, 14–16), presumably due to environmental parameters such as host density (17) and mucosal tissue structure (18). Phage integration into animal-associated bacterial genomes (i.e., prophage) can alter the phenotype of the host bacterium through lysogenic conversion (19, 20), as well as enhance biofilm formation and thereby horizontal gene transfer among co-occurring bacteria (21, 22). The prevalence of temperate phages in host-associated microbiomes suggests that these phages may more intimately evolve with their bacterial hosts and/or shape the composition of the bacterial communities. Additionally, the discovery of intraspecific and interspecific core viromes dominated by phages across animal systems is often reflective of the core bacterial communities described in these same organisms (11, 23–25). Although it has been suggested previously (24), phylosymbiosis at the viral level has yet to be explicitly demonstrated, and evidence for this tripartite association pattern could underpin new ecological and functional interactions between an animal host, its bacterial community, and the viruses infecting both.

## RESULTS

### Virome samples and assemblies.

Viral purifications from adults of three species of *Nasonia*, a *Nasonia* introgression line, and the Mediterranean flour moth Ephestia kuehniella were sequenced. Each of the pure *Nasonia* species (N. vitripennis, N. longicornis, and N. giraulti) maintains their natural *Wolbachia* infections from supergroup A. The introgression line IntG has the genome of N. giraulti and the cytoplasm of N. vitripennis, including the maternally inherited supergroup A *Wolbachia* strain *w*VitA from N. vitripennis (26). E. kuehniella harbors a supergroup B *Wolbachia* strain named *w*CauB (27). Viral particle sequencing and single sample assembly statistics are outlined in Table S1 in the supplemental material.

10.1128/mSystems.00131-18.2 TABLE S1 Assembly statistics. Download Table S1, PDF file, 0.05 MB. Copyright © 2018 Leigh et al. 2018 Leigh et al. This content is distributed under the terms of the Creative Commons Attribution 4.0 International license.

### Phylosymbiosis: host relatedness reflects compositional similarity of viral metagenomes.

Phylosymbiosis describes a significant host phylogenetic signal on host-associated microbiome communities (1). Bacterial communities frequently, but not universally, exhibit this relationship under wild and laboratory conditions (1, 7). For viromes, there is no *a priori* reason to expect that phylosymbiosis will occur because inducible proviruses and/or lytic viruses, i.e., the targets of this study, may constitute a small subset of the total viral DNA in bacterial and eukaryotic genomes, and active viral particles have the potential to lyse and shift bacterial communities that may disrupt phylosymbiosis. Here we evaluate if the *Nasonia* viromes form phylosymbiotic community relationships.

**FIG 1:**
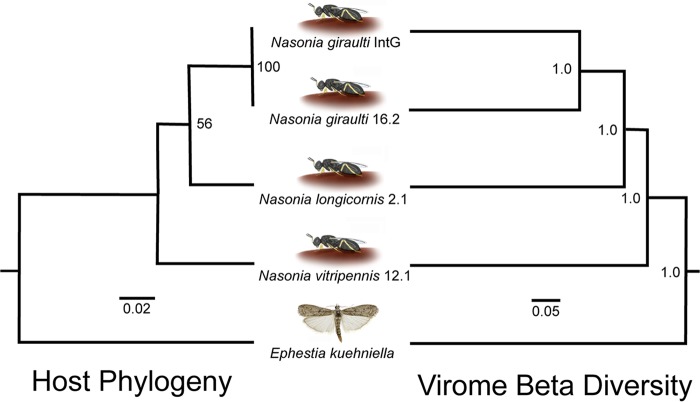
Phylosymbiosis occurs between insects and their viral communities. The host phylogeny is constructed with PHYML from 385 bp of the cytochrome oxidase I gene, and the UPGMA hierarchical cluster relationships of the viromes are based on Bray-Curtis beta diversity distances. Significance of topological congruence was determined using a previously described method (1) based on the rooted Robinson-Foulds (*P* value: 0.00451) and rooted matching cluster (*P* value: 0.00451) metric with a total of 100,000 randomized topologies simulating a null hypothesis of stochastic virome ecological assembly.

The phylogeny of *Nasonia* spp. rooted with the outgroup E. kuehniella is based on DNA sequences of the cytochrome oxidase I (COI) gene as previously shown (1, 7, 28–31). It resulted in the same branching pattern as the dendrogram generated from Bray-Curtis beta diversity of the viral metagenomes across the host species (Fig. 1). The matching cluster and Robinson-Foulds tree metrics were utilized to calculate host phylogenetic and virome dendrogram topological congruence, which is highly significant based on both metrics with 100,000 randomly bifurcating trees to simulate stochastic virome assembly (1) (*P* value = 0.00451). Additionally, using the same methodology, matching cluster and Robinson-Foulds metrics were evaluated by the Binary Jaccard beta diversity index, which produced identical results using viral presence and absence within each sample. Taken together, these findings comprise one of the first lines of evidence for phylosymbiosis in host-associated viral communities. We next evaluated the number and types of viruses that comprise these phylosymbiotic communities.

### Characterizing the *Nasonia* virome: host genetic effects, the virome core, and toxins.

**FIG 2:**
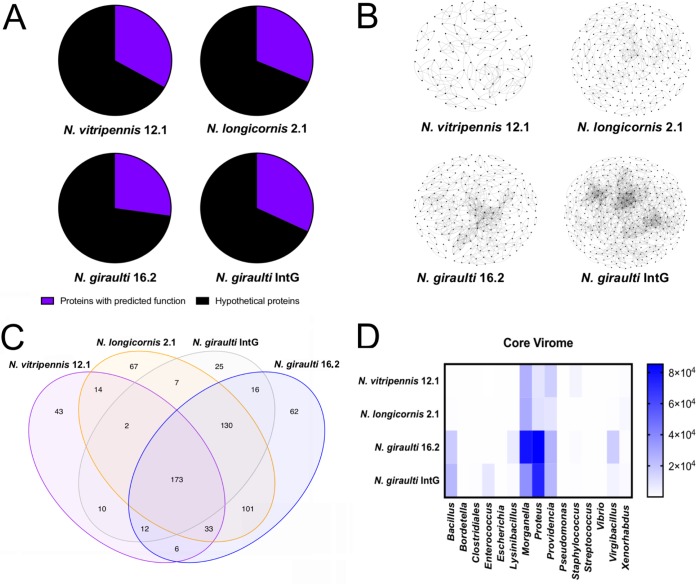
*Nasonia* species harbor a modest core virome. (A) Percent viral contigs with at least one functionally annotated gene as determined by Pfam analysis. (B) Viral protein cluster analysis illustrating diversity of viral proteins within each virome. Each dot represents a unique viral protein and connecting lines indicate >70% sequence similarity between two proteins. (C) Venn diagram illustrating the viral contigs unique within and shared between the *Nasonia* species. (D) Taxonomic affiliation of the 219 members of the identified core virome as determined by BLASTx against the nr database. Shading indicates the relative abundance of each member within single viromes and was determined by read mapping to viral contigs.

Unlike many environmental viral metagenomes, the majority of the viral contigs from the insects studied here had at least one gene with BLASTx similarity to either known lytic viruses or genes from their potential respective hosts. An average of 30.9% of the genes identified in each of the samples have a predicted annotation and function (Fig. 2A). Therefore, to identify groups of proteins independent of the database annotations, unique protein clusters, defined as groups of proteins with significant sequence similarity (>70%), were determined in each of the samples by the protein clustering tool vContact (Fig. 2B). The protein cluster networks identified N. giraulti and IntG as the most diverse viromes, which share a N. giraulti genetic background but vary in the origin of their cytotype. This result suggests that host genotype rather than cytotype more strongly impacts diversity of the host-associated viral metagenome, either through interactions with phage directly or through interactions with the bacteria harboring these phages. N. longicornis and N. vitripennis yielded approximately 50% fewer unique protein groups in their viromes.

To assess identity and diversity of proteins with predicted function in the viromes, contigs from all samples were compared to the protein family (Pfam) database. Each *Nasonia* species virome maintained a small, host-specific set of Pfams ranging from 6.7% to 14.8% of the Pfams (Table S2). Precisely 24.4% of the Pfams (173) were shared among all of the *Nasonia* samples, which parallels the 21% of the total contigs described as the core virome below. Across all species, the most abundant Pfam (4.7% of total Pfam predictions) was the helix-turn-helix (HTH) DNA-binding motif (PF01381) followed by the phage integrase Pfam (PF00589, 2.9% of total Pfam predictions).

10.1128/mSystems.00131-18.3 TABLE S2 Pfam assignments in viral metagenomes. Download Table S2, PDF file, 0.3 MB. Copyright © 2018 Leigh et al. 2018 Leigh et al. This content is distributed under the terms of the Creative Commons Attribution 4.0 International license.

To further explore the protein content of the viromes and the interactions that could underpin phylosymbiosis between hosts and their viromes, we assessed if domains similar to known toxins or domains that interact with eukaryotic hosts were present in these viruses using the Pfam annotations. Proteins identified as toxins and eukaryotic-interacting domains span immunoglobulin peptidases, virulence genes, lysins, and others (indicated by boldface in Table S2). Domains identified within these groups were found in viral contigs isolated from N. giraulti and IntG where 36 and 34 unique identifiable toxin and eukaryotic-interacting proteins spanned 0.045% and 0.067% of the total contigs, respectively. N. vitripennis and N. longicornis maintained 17 and 25, which spanned 0.025% and 0.098% of the contigs, respectively. One identified domain is the hemolysin-encoding XhlA (PF10779) detected in *Bacilli* class-associated contigs in all of the samples, which was also the most abundant in the N. giraulti and the IntG introgression samples. This family of hemolysins, first observed in the entomopathogenic Xenorhabdus nematophila, notably lyses insect immune cells (32).

Next, core viral contigs shared among all samples were determined by read mapping to the assembled contigs using the iVirus pipeline (33). Across the *Nasonia* samples, the core was comprised of 219 viral contigs or 21% of total *Nasonia* viral contigs (Fig. 2C). Of these core viral contigs, the majority (84%) are homologous to members infecting species of the most abundant bacterial genera found within the *Nasonia* gut microbiome: *Morganella*, *Proteus*, and *Providencia* (Fig. 2D). Additionally, 14 of the core viral contigs are homologous to sequences from the *Bacilli* class, all of which are relatively more abundant in N. giraulti and IntG. Two core viral contigs showed amino acid similarity to sequences in the genome of the entomopathogenic Xenorhabdus innexi (34); they contain phage structural genes typical of active phage particles. Additionally, the complete genome of *w*VitA phage WO, a prophage of the obligate intracellular bacterium *Wolbachia* that infects each of these aforementioned *Nasonia* species (35), was detected only in N. vitripennis. The genome of this prophage was described previously (36) and produces viral particles as seen in transmission electron microscopy in N. vitripennis (35).

### Viral diversity among *Nasonia* species.

**FIG 3:**
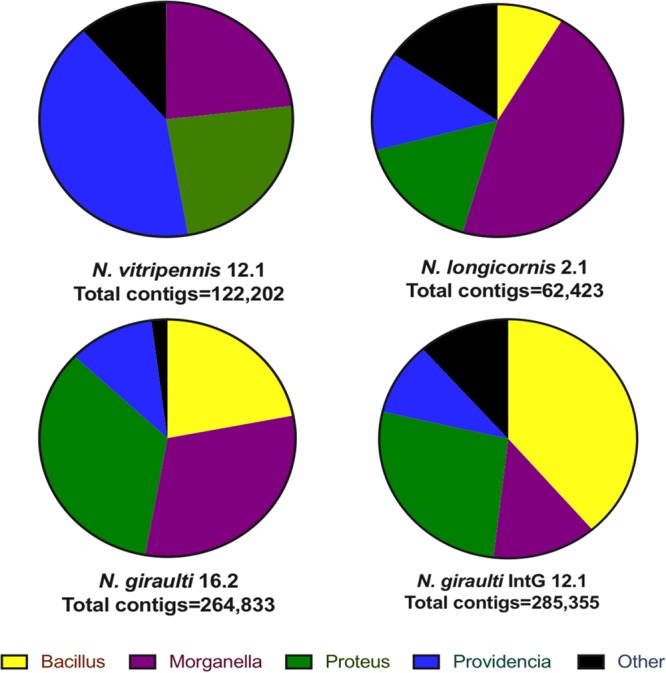
Viral communities are distinguishable between *Nasonia* species and dominated by a few taxa. The assigned taxa are bacterial genera that harbor sequences, presumably prophages, homologous to the viral protein sequences. The relative abundance of viral contigs within each species is variable. Taxonomy is determined by highest similarity through tBLASTx against the nr database.

The number of reads mapped to each viral contig adjusted for contig size varied among species, highlighting distinct relative abundance differences of *Proteus*, *Providencia*, *Morganella*, and *Bacilli* phages among the *Nasonia* species (Fig. 3). *Proteus* phages dominate the N. giraulti virome at 34.3% of the total contigs, and *Morganella* phages make up the next largest portion at 30.8%. *Morganella* phages dominate the N. longicornis virome at 45.9%, and *Providencia* dominates the N. vitripennis virome at 41.4%. Phages with similarity to the *Bacillaceae* family outnumber all other groups in introgression line IntG (38.7%), followed by *Proteus* (26.8%). Thus, a different family of phages dominates each individual host genotype as was similarly shown for bacterial communities associated with these wasps (1). For example, *Providencia* bacteria dominate the N. vitripennis microbiome (1), which correlates with the highest abundance of *Providencia* phages in the sequenced virome.

### Complete and abundant viral genomes.

**FIG 4:**
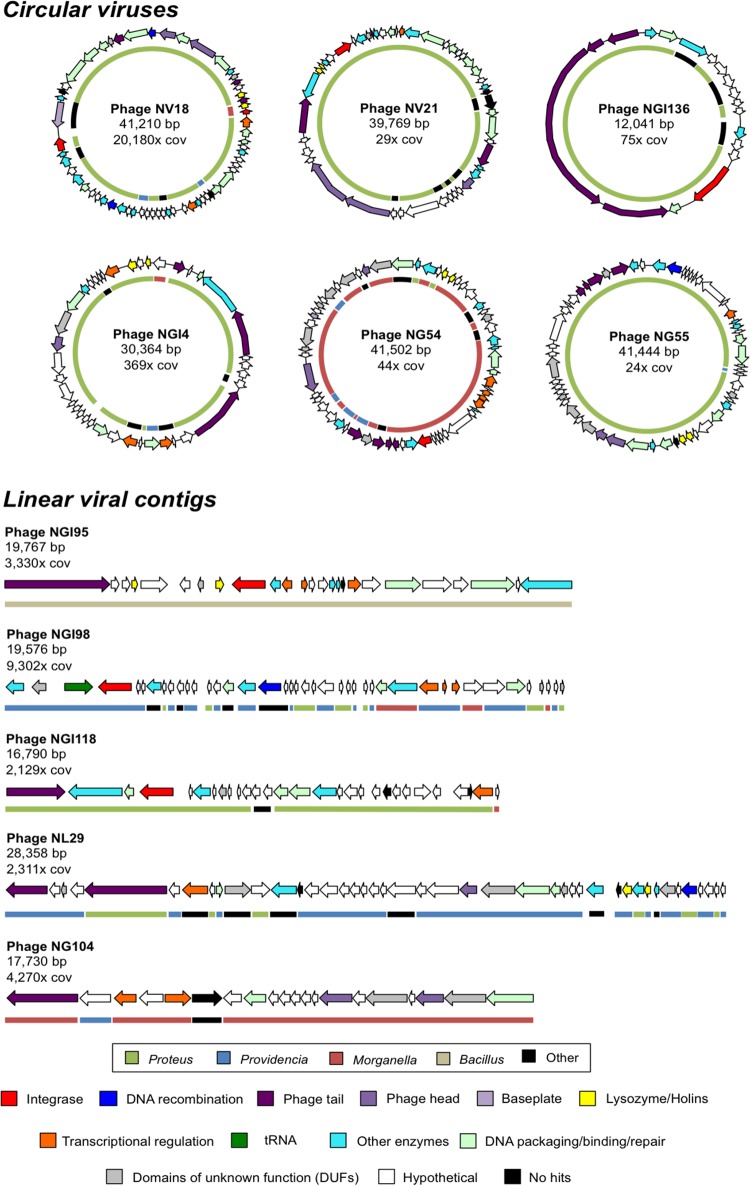
Taxonomy and annotated function of circular genomes and most abundant linear viral contigs in *Nasonia*. Six complete and circular viral genomes were part of the core *Nasonia* virome, five of which consisted mostly of open reading frames (ORFs) with similarity to *Proteus* proteins as determined via BLASTx through NCBI against the nr database (denoted by colored line of inner circles). A total of six viral contigs (circular phage NV18 and five linear contigs above) composed >50% of reads from each of the samples. Each of these dominant viruses was shared among all of the samples with the exception of phage NGI95, which was present only in N. giraulti and N. giraulti IntG. Colored arrows indicate predicted gene function, and colored inner circles represent the genus of the closest BLASTx hit of each gene to the nr database.

Six putative circular phage genomes in the core virome with moderate amino acid similarity (>70% homology) to sequences in members of *Proteus* and *Morganella* were identified using the viral classification program VirSorter and annotated using BLASTx against the RefSeq database (Fig. 4). None of the circular phage genomes were previously reported as prophages in the bacterial genomes from which they were identified, nor have they been previously described as forming lytic phage particles. Genes in five of these circular phage particle genomes have closest matches in the *Proteus* bacterial genus. The other, phage NG54, contains genes most homologous to *Morganella* spp. Thus, these six newly assembled phage genomes, as well as most contigs recovered here, establish the hypothesis that homologous regions in close bacterial relatives of those that colonize *Nasonia* are prophages with the potential to form phage particles.

To determine the most abundant phage variants within the *Nasonia* virome, reads were mapped to each of the viral contigs, and six contigs with total read coverage over 2,000× were identified as the most abundant. These six phage genomes, one of which was circular (phage NV18, Fig. 4) and five of which were incomplete genomes (contigs), represented 26% of the total reads in N. longicornis and over 50% of reads in the other three samples. Five of these six most abundant phages were dominated by ORFs with similarity to *Proteus*, *Providencia*, and *Morganella* as well. However, phage NGI95 (Fig. 4) shared the most similarity with *Bacilli* proteins and was detected only in the introgression line IntG and N. giraulti. Again, a large number of these genes encode unannotated hypothetical proteins, and of these abundant linear viral contigs, three of the five maintain identifiable integrase genes.

Last, two additional novel circular phage genomes recovered from N. vitripennis (phage NV11X) and N. giraulti (phage NG24X) are composed of *Xenorhabdus* genes, have 94% nucleotide similarity to each other, and maintain predicted phage structural and hypothetical proteins (Fig. S1). These two *Xenorhabdus* phages show an average of 64% amino acid identity and complete genome synteny to predicted proteins of Xenorhabdus innexi and KK7.4, suggesting that prophages are present within these two bacterial genomes. *Xenorhabdus* bacteria are insect pathogens that suppress the immune system and produce numerous virulence factors such as hemolysin and cytotoxin that result in insect lethality (37–39). Although hemolysins were found in these viromes, they were associated with *Bacilli* phages and not these *Xenorhabdus* phages, consistent with previous reports that the *Xenorhabdus* bacteria themselves encode these toxins (37–39).

10.1128/mSystems.00131-18.1 FIG S1 Complete *Xenorhabdus* phage genomes. Two similar (94% nucleotide similarity) circular *Xenorhabdus* phage genomes were recovered, one from *N. vitripennis* (phage NV11X) and the other from *N. giraulti* (phage NG24X). Download FIG S1, PDF file, 0.05 MB. Copyright © 2018 Leigh et al. 2018 Leigh et al. This content is distributed under the terms of the Creative Commons Attribution 4.0 International license.

## DISCUSSION

Phylosymbiosis between host and bacterial communities is emerging as a trend in microbiome studies of the animal world, across both vertebrate and invertebrate species (1, 40–42). While the genetic and biochemical mechanisms underlying phylosymbiosis require more study, animal performance or fitness is often highest when animals contain a homospecific microbiome in comparison to a heterospecific microbiome (1, 43). These findings imply that there are mechanisms by which animals differentially respond to the membership of the microbiome and/or vice versa. Animal-associated viromes, often composed of mostly phages, have generally received much less study than bacterial microbiomes, and there is no *a priori* reason to expect that phylosymbiosis will occur in phage metagenomes because animals are not expected to directly exert influence on membership, nor is the phage community expected to directly determine which animal it occurs in. However, evidence for direct phage protein interactions within insect hosts is found in endosymbionts where a stable association among the phage, bacterium, and animal has been established (44–46). The bacterial endosymbionts of *Nasonia*, *Wolbachia* and its prophage (WO), represent another potential case as the phage-encoded Cif proteins cause (47) and rescue (48) reproductive parasitism phenotypes in arthropod hosts. Additionally, phage particles can bind animal mucus on epithelial tissues via immunoglobulin domains found on the surface of some phage capsids, providing a form of immunity against colonizing bacteria (49, 50). The phages in this environment can also be transcytosed across the epithelial membrane and trafficked through the Golgi apparatus via the endomembrane system (51), further highlighting a direct interaction between phages and animals.

While bacteriophages may simply exhibit phylosymbiosis in a passive manner by association with phylosymbiotic bacterial communities, inducible prophages and/or lytic phages that are the subject of study here may only constitute a small subset of the phage DNA in bacterial genomes. Moreover, active phage particles have the potential to lyse and shift bacterial communities that may disrupt phylosymbiosis. Thus, there is no preferred reason to expect the metagenome of the purified community of virus particles will exhibit phylosymbiosis. Similarly to other animal viromes (8, 10–12, 14, 24), the majority of viruses within *Nasonia* species are phages, and they appear to be derived mainly from prophages predicted in the most prevalent bacterial genera in *Nasonia*: *Proteus*, *Providencia*, and *Morganella*. Previous reports in *Hydra* also showed that viromes were host species specific, composed mostly of phages, and partially phylosymbiotic, although congruence of the host and virome topologies was not investigated (24). Interestingly, wild-caught and lab strains of the same species (Hydra vulgaris) harbor significantly different bacterial communities (52, 53) and therefore maintained unique viral communities as well (24).

Here we describe the first report of phylosymbiosis among host-associated viromes in the parasitoid wasp genus *Nasonia*. Members of this genus diverged very recently, between 200,000 and 1 million years ago (31), and controlled rearing of each species leads to distinguishable, phylosymbiotic microbiomes that significantly impact development and survival (1, 7). Indeed, interspecific microbiota transplantation causes 25 to 42% decreases in *Nasonia* survival to adulthood compared to intraspecific microbial transplantations (1). Moreover, hybrid death in the F2 generation is due to a breakdown in phylosymbiosis whereby inoculations of resident gut bacterial species into germfree hybrids recapitulate hybrid lethality (7).

The results here are consistent with the model that if bacterial communities show phylosymbiosis with animal hosts, so too will their viromes. More simply put, viral phylosymbiosis appears to emerge as a by-product of host-bacterium phylosymbiosis. From a methodological perspective, the result is striking given that the sequencing methods to build the bacterial and viral community dendrograms are fundamentally different: 16S amplicon sequencing versus shotgun viral metagenomics. Machine learning on 16S amplicon data previously specified that three of the major distinguishing bacterial genera in *Nasonia* are closely related symbionts from the *Enterobacteriaceae* family (genera *Proteus*, *Providencia*, and *Morganella*) (1). Interestingly, abundant phages of *Proteus*, *Providencia*, and *Morganella* dominate the virome identified within all of the pure *Nasonia* species (Fig. 2D and Fig. 3). Nonetheless, distinguishability of the viromes between *Nasonia* species is evident through at least two observations: (i) one of the most abundant viruses, phage NGI95, is solely found in the Nasonia giraulti genotype and (ii) the majority of the phage particle genetic diversity within N. giraulti and IntG is represented by a shared group of abundant *Bacillaceae* phages (Fig. 2B and C). Similarities between the samples with an N. giraulti genetic background support the hypothesis that host genotype, rather than cytotype, plays a role in shaping elements of the phage community structure.

Many of the dominant bacteria present within *Nasonia* are related to well-studied human pathogens present in enteric diseases (54–60) in addition to other insects (61–63), and genomes are therefore available (64–66). However, most prophage genomes present within these bacteria have not yet been described, and 69% of the genes remain annotated as encoding hypothetical proteins. Thus, the majority of the viruses found in this study were active, unannotated phages of the most prevalent types of bacteria found in *Nasonia*.

We assembled five complete *Proteus* phages and one *Morganella* phage (Fig. 3). Four of these phages (phages NGI4, NV18, and NG55 [*Proteus*] and phage NG54 [*Morganella*]) maintained an integrase gene, indicating likely integration into their host’s genome as a prophage. One of the circular *Proteus* phages maintaining an integrase, phage NV18, was by far the most prevalent phage in all of the samples with over 20,000-fold read coverage from N. vitripennis compared to the 10- to 200-fold coverage of most other viral contigs. This phage genome is composed of mostly hypothetical proteins and proteins with domains of unknown function. Many of these phages show amino acid similarity to sequences within the *Proteus*, *Providencia*, and *Morganella* genera (Fig. 3). These similarities suggest that the described phages may be able to infect members across these sister genera, integrating and acquiring or leaving behind genes in the process.

The discovery of animal-bacterial-viral phylosymbiosis provides a new insight into the tritrophic relationships between animal evolution, bacterial communities, and their phage communities. We note that phylosymbiosis does not equate to coevolution, codiversification, or cospeciation because these are evolutionary processes that assume divergence from a common ancestor. Phylosymbiosis is an eco-evolutionary pattern whereby ecological similarities in the microbiome, or virome in this case, parallel phylogenetic relationships of the host. These patterns are not necessarily ones that occur long term, and they can change rapidly in time or space. However, the detection of phylosymbiosis of the virome is consistent with host identity providing either a direct or indirect influence that partitions clustering relationships of viral particle communities in a manner that reflects animal evolution among closely related species. Whether these patterns hold in wild populations will require future study.

The microbiome has now been widely recognized as a key component of many animal functions, and alterations of this bacterial community can result in performance or fitness reductions (67–69). Prophages are more common than lytic phages in stable host-associated microbial communities (8, 10), outnumber bacteria ∼3:1, and represent a potential structural force for establishment and maintenance of a microbiome (70–72). Intimate associations among phages, bacteria, and their animal hosts are complex, and further studies investigating phylosymbiotic phage communities throughout the animal kingdom are necessary to gain a fuller understanding of the role that microbiomes and viromes play in animal functions and evolution.

## MATERIALS AND METHODS

### Sample collection and sequencing.

*Nasonia* species were reared as previously described (7). Four strains were used in this study: Nasonia vitripennis (strain 12.1), N. longicornis (2.1), N. giraulti (16.2), and N. giraulti (IntG 12.1). Each strain maintains *Wolbachia* infections of the A supergroup. The IntG line was generated by repeatedly backcrossing N. vitripennis 12.1 females to uninfected N. giraulti RV2R males for nine generations to generate a line that contains *w*VitA-infected cytoplasm of N. vitripennis in the genetic background of N. giraulti (26). Each strain was maintained under constant light at 25°C and raised on flesh fly pupae (Sarcophaga bullata). The transfected line of the Mediterranean flour moth Ephestia kuehniella harboring *Wolbachia* strain *w*CauB was obtained from Takema Fukatsu and Tetsuhiko Sasaki (27). Moths were maintained at 24°C and 70% humidity on a diet consisting of wheat bran, glycerol, and dried yeast (20:2:1 [wt/wt]).

Whole insects were suspended in sterile SM buffer and homogenized to release the viruses from the animal tissue. Viral particles were PEG precipitated as previously described (36) and filtered through an 0.22-μm filter. Viral DNA was extracted using the Qiagen MinElute Virus Spin kit, amplified using the Qiagen REPLI-g minikit, and sequenced on the Illumina HiSeq 2000 platform with paired-end reads (2 × 100 bp).

### Bioinformatics.

Mate-pair reads from the viromes were analyzed using the iVirus pipeline (33). First, the sequences were trimmed using Trimmomatic 0.35.0 (73) and quality checked using FastQC. *De novo* assembly of mate-pair reads was completed using SPAdes 3.6.0 (74) with a k-mer value of 63 and default parameters. Assembly quality was determined by QUAST (75) and is reported in Table S1 in the supplemental material. All samples were coassembled with SPAdes 3.6.0 with a k-mer of 63 to generate a single reference file and run through VirSorter (76) in addition to the single assemblies. Viral contigs less than 500 bp and with coverage of less than five were removed from further analysis. Reads were then mapped back to the VirSorter viral contig outputs to estimate the relative abundance of each viral contig for each sample. BowtieBatch (33) was used to run bowtie2 on all samples of the coassembled contigs and produced BAM output files read by Read2RefMapper to generate relative abundance and coverage plots for each viral contig within each metagenome. To consider a contig present within an individual sample, reads from that sample needed to cover 75% of the viral contig from the coassembled virome. Venn diagrams were generated using the VennDiagram package (77) through R v 3.3.2 software to display the overlap of contigs in different gut compartments using the mapping data from Read2RefMapper (33). Relative abundance plots were illustrated using GraphPad’s Prism v 7.0c. Viral protein cluster diversity was determined using vContact through the iVirus pipeline (33, 78) and visualized using Gephi 0.9.2 (79).

The VirSorter output for single assemblies was used for taxonomic classification against the NCBI nr protein database. All taxonomic classifications were determined using the top BLASTx hit, with a threshold score of 50 on BLAST bitscore. Open reading frames (ORFs) were predicted and annotated using Prokka v1.12.0 (80). Additionally, protein families (PFAMs) within the ORFs were identified with InterProScan v5.26.65 (81). jModelTest v2.1.7 (82) was performed to determine the optimal model of host gene evolution and, using this model (PHYML with the JC69 substitution model), a phylogenetic tree was constructed for the host species (as previously described in reference 1) from a nucleotide alignment of the mitochondrial cytochrome oxidase subunit I (COI) genes. Virome similarities as determined by Bray-Curtis beta diversity unweighted pair group method with arithmetic mean (UPGMA) clustering were determined using read coverage counts of viral contigs. These count profiles were rarefied 10 times to a depth of 16,400 counts for each host virome to normalize for differential sequencing coverage. Bray-Curtis beta diversity and resulting UPGMA clustergrams between host viromes were calculated, and UPGMA trees were averaged to generate a consensus clustergram across the rarefied community profiles. Phylosymbiosis as measured through topological similarity between the host phylogeny and the virome clustergram was evaluated using the rooted Robinson-Foulds and rooted matching cluster methods previously described (1). Significance was determined by comparing the observed degree of congruence to the congruence obtained across 100,000 randomized tree topologies using a custom script with methods previously described (1).

### Data accessibility.

Assembled contigs from each viral metagenome have been submitted to the WGS database of NCBI under BioProject PRJNA481165. Additionally, each circular genome has been submitted to the NCBI nr database under the accession numbers MK047638 to MK047643.
