# Supplementary material for: Finer-Scale Phylosymbiosis: Insights from Insect Viromes
Source: mSystems. 2018 Dec 18;3(6):e00131-18. doi: 10.1128/mSystems.00131-18 (PMC6299154; doi:10.1128/mSystems.00131-18)
Supplement: TABLE S1 [file sys006182303st1.pdf]

| | *N. vitripennis* 12.1 | *N. longicornis* 2.1 | *N. giraulti* IntG | *N. giraulti* 16.2 |
|---|---|---|---|---|
| # reads (bp) | **55,018,874** | **16,967,060** | **54,305,634** | **51,603,058** |
| # contigs (>=0 bp) | 5,338 | 10,288 | 11,154 | 7,625 |
| # contigs (>=1000 bp) | 658 | 961 | 1,677 | 1,060 |
| # contigs (>=5000 bp) | 136 | 198 | 352 | 241 |
| # contigs (>=10000 bp) | 82 | 105 | 191 | 146 |
| # contigs (>=25000 bp) | 34 | 36 | 75 | 83 |
| # contigs (>=50000 bp) | 13 | 6 | 23 | 36 |
| Total length (>=0 bp) | 6,233,827 | 7,400,287 | 14,313,288 | 10,974,448 |
| Total length (>=1000 bp) | 4,574,682 | 4,746,396 | 10,687,227 | 8,488,860 |
| Total length (>=5000 bp) | 3,539,370 | 3,281,465 | 8,066,250 | 6,923,197 |
| Total length (>=10000 bp) | 3,164,282 | 2,636,751 | 6,950,497 | 6,263,109 |
| Total length (>=25000 bp) | 2,380,066 | 1,550,630 | 5,146,661 | 5,246,785 |
| Total length (>=50000 bp) | 1,631,890 | 488,296 | 3,224,513 | 3,479,271 |
| # contigs | 1,478 | 2,247 | 3,730 | 2,388 |
| GC (%) | 40.57 | 42.08 | 39.73 | 39.63 |
| N50 | 21,830 | 8,245 | 16,642 | 34,618 |
| N75 | 3,076 | 1,710 | 2,686 | 4,157 |
| L50 | 42 | 125 | 120 | 65 |
| L75 | 219 | 545 | 629 | 269 |
| #N's per 100 kbp | 0 | 0 | 0 | 0 |
