# Supplementary material for: Finer-Scale Phylosymbiosis: Insights from Insect Viromes
Source: mSystems. 2018 Dec 18;3(6):e00131-18. doi: 10.1128/mSystems.00131-18 (PMC6299154; doi:10.1128/mSystems.00131-18)
Supplement: TABLE S2 [file sys006182303st2.pdf]

Pfam	ID	N_Vit	N_Gir	N_long	IntG
pf01381	(HTH_3)	24	93	39	186
pf00589	(Phage_integrase)	15	75	26	98
pf05709	(Sipho_tail)	2	37	16	72
pf05065	(Phage_capsid)	9	19	11	46
pf06114	(Peptidase_M78)	3	31	6	46
pf00717	(Peptidase_S24)	13	20	17	34
pf01844	(HNH)	4	23	9	44
pf05135	(Phage_connect_1)	8	19	9	39
pf04860	(Phage_portal)	9	15	9	38
pf06605	(Prophage_tail)	3	25	14	46
pf14659	(Phage_int_SAM_3)	4	26	8	42
pf00436	(SSB)	6	20	9	32
pf03796	(DnaB_C)	3	14	8	30
pf04883	(HK97-gp10_like)	6	22	7	31
pf10145	(PhageMin_Tail)	5	24	9	28
pf00574	(CLP_protease)	4	11	6	26
pf01510	**(Amidase_2)**	3	26	11	42
pf13443	(HTH_26)	0	17	10	28
pf01555	(N6_N4_Mtase)	4	14	9	23
pf01695	(IstB_IS21)	7	18	8	30
pf09643	(YopX)	0	17	5	27
pf00271	(Helicase_C)	2	10	7	25
pf03354	(Terminase_1)	8	14	6	38
pf14657	(Arm-DNA-bind_4)	1	19	6	30
pf05521	(Phage_H_T_join)	6	9	7	23
pf13392	(HNH_3)	3	10	9	21
pf00692	(dUTPase)	1	9	4	17
pf03374	(ANT)	1	15	5	22
pf12844	(HTH_19)	1	15	4	16
pf13479	(AAA_24)	2	12	7	19
pf01520	**(Amidase_3)**	3	17	4	23
pf05866	(RusA)	9	16	3	24
pf02368	**(Big_2)**	6	13	9	31
pf08281	(Sigma70_r4_2)	0	12	2	16
pf00196	(GerE)	0	2	2	7
pf01551	(Peptidase_M23)	3	13	5	18
pf01471	(PG_binding_1)	0	9	4	14
pf04630	(Phage_TTP_1)	2	10	6	21
pf05133	(Phage_prot_Gp6)	0	23	7	29
pf12363	(Phage_TAC_12)	0	11	5	18
pf08863	(YolD)	0	15	4	23
pf04688	(Holin_SPP1)	0	10	5	24
pf00772	(DnaB)	2	7	4	17
pf11753	(DUF3310)	3	9	5	22
pf02867	(Ribonuc_red_lgC)	0	10	8	12
pf06199	(Phage_tail_2)	0	12	6	19
pf00239	(Resolvase)	2	12	6	20
pf13730	(HTH_36)	5	11	5	18
pf02498	(Bro-N)	1	10	4	16
pf03819	(MazG)	0	5	6	16
pf09669	(Phage_pRha)	2	11	5	14
pf13884	(Peptidase_S74)	0	8	3	17
pf04233	(Phage_Mu_F)	6	19	7	17

pf05105	(Phage_holin_4_1)	1	13	3	22
pf08761	(dUTPase_2)	1	18	4	17
pf00583	(Acetyltransf_1)	0	1	0	0
pf01580	(FtsK_SpoIIIE)	0	8	3	18
pf03864	(Phage_cap_E)	1	12	4	17
pf04586	(Peptidase_S78)	4	6	6	16
pf00268	(Ribonuc_red_sm)	0	9	6	10
pf03837	(RecT)	5	17	6	21
pf04851	(ResIII)	1	5	3	14
pf00145	(DNA_methylase)	4	6	5	16
pf13102	(Phage_int_SAM_5)	2	12	8	12
pf00877	(NLPC_P60)	8	3	5	9
pf03592	(Terminase_2)	4	19	5	20
pf10552	(ORF6C)	2	11	1	13
pf16079	(Phage_holin_5_2)	0	9	6	16
pf07508	(Recombinase)	1	8	4	16
pf13302	(Acetyltransf_3)	0	1	0	0
pf01464	(SLT)	2	9	2	8
pf04466	(Terminase_3)	1	16	5	17
pf05037	(DUF669)	1	4	3	13
pf06152	(Phage_min_cap2)	0	12	4	19
pf09524	(Phg_2220_C)	2	5	0	13
pf00149	(Metallophos)	2	5	2	5
pf00313	(CSD)	1	1	1	3
pf01391	**(Collagen)**	0	4	2	13
pf04865	(Baseplate_J)	5	13	3	10
pf07261	(DnaB_2)	1	11	1	8
pf07410	(Phage_Gp111)	0	6	3	6
pf08774	(VRR_NUC)	0	3	4	12
pf09588	(YqaJ)	2	8	6	15
pf13128	(DUF3954)	0	4	0	9
pf00593	(TonB_dep_Rec)	0	1	0	0
pf01926	(MMR_HSR1)	0	0	0	2
pf02562	(PhoH)	0	5	5	9
pf07715	(Plug)	0	1	0	0
pf12728	(HTH_17)	0	6	2	15
pf00476	(DNA_pol_A)	1	7	6	13
pf04404	(ERF)	3	8	2	14
pf07463	(NUMOD4)	0	2	3	11
pf08241	(Methyltransf_11)	0	2	0	3
pf10665	(Minor_capsid_1)	0	4	2	13
pf10779	**(XhlA)**	1	5	2	8
pf13155	(Toprim_2)	0	8	6	12
pf13814	(Replic_Relax)	0	5	2	16
pf01476	**(LysM)**	1	7	2	4
pf01497	(Peripla_BP_2)	0	1	0	0
pf04531	(Phage_holin_1)	0	7	3	12
pf06725	**(3D)**	0	7	3	11
pf12796	(Ank_2)	8	0	0	0
pf17288	(Terminase_3C)	1	14	5	15
pf03288	(Pox_D5)	0	2	1	9
pf04542	(Sigma70_r2)	0	4	3	6
pf08706	(D5_N)	0	1	2	9
pf11114	(Minor_capsid_2)	0	5	2	13

pf11367	(DUF3168)	0	6	2	10
pf12691	(Minor_capsid_3)	0	4	2	13
pf13539	(Peptidase_M15_4)	4	8	4	9
pf01612	(DNA_pol_A_exo1)	0	8	5	10
pf02075	(RuvC)	0	4	4	9
pf07083	(DUF1351)	1	9	3	10
pf00847	(AP2)	1	7	4	5
pf03477	(ATP-cone)	0	5	3	8
pf05595	(DUF771)	0	2	1	7
pf08346	(AntA)	0	5	1	10
pf13476	(AAA_23)	0	3	1	9
pf13481	(AAA_25)	3	4	0	5
pf00176	(SNF2_N)	1	6	2	10
pf00317	(Ribonuc_red_lgN)	0	5	3	4
pf07352	(Phage_Mu_Gam)	0	3	2	10
pf12706	(Lactamase_B_2)	0	9	2	9
pf17289	(Terminase_6C)	5	10	3	10
pf00082	(Peptidase_S8)	0	3	5	6
pf04545	(Sigma70_r4)	0	5	2	4
pf05119	(Terminase_4)	4	3	1	10
pf06356	(DUF1064)	0	8	3	9
pf08230	**(CW_7)**	0	6	5	11
pf09851	(SHOCT)	0	3	1	9
pf00216	(Bac_DNA_binding)	0	5	1	3
pf00258	(Flavodoxin_1)	0	1	1	0
pf11799	(IMS_C)	0	9	0	6
pf13356	(Arm-DNA-bind_3)	4	6	2	6
pf13408	(Zn_ribbon_recom)	1	7	1	8
pf00303	(Thymidylat_synt)	0	5	4	4
pf00578	**(AhpC-TSA)**	0	0	0	1
pf00665	(rve)	0	1	1	3
pf00753	(Lactamase_B)	0	2	0	3
pf02086	(MethyltransfD12)	3	4	2	4
pf02899	(Phage_int_SAM_1)	1	2	2	5
pf03237	(Terminase_6)	5	11	4	9
pf03279	(Lip_A_acyltrans)	0	0	0	1
pf05257	**(CHAP)**	3	4	4	5
pf06530	(Phage_antitermQ)	4	2	1	4
pf06810	(Phage_GP20)	0	7	3	11
pf06854	(Phage_Gp15)	0	5	1	10
pf12684	(DUF3799)	4	7	1	5
pf13022	(HTH_Tnp_1_2)	0	5	2	7
pf13412	(HTH_24)	1	1	1	1
pf14265	(DUF4355)	0	9	1	10
pf00132	(Hexapep)	0	1	2	1
pf00959	(Phage_lysozyme)	5	6	5	4
pf03245	(Phage_lysis)	6	6	6	4
pf08858	(IDEAL)	0	2	3	6
pf00929	(RNase_T)	1	1	1	1
pf01832	(Glucosaminidase)	0	1	1	2
pf05100	(Phage_tail_L)	2	2	4	4
pf05488	(PAAR_motif)	1	0	0	0
pf05939	(Phage_min_tail)	2	2	4	3
pf06378	(DUF1071)	0	2	1	4

pf09681	(Phage_rep_org_N)	0	5	1	8
pf10651	(DUF2479)	2	6	2	8
pf13274	(DUF4065)	0	5	0	2
pf13361	(UvrD_C)	0	4	1	3
pf13411	(MerR_1)	0	1	0	0
pf13560	(HTH_31)	0	2	0	8
pf14464	(Prok-JAB)	3	2	2	5
pf14520	(HHH_5)	0	1	0	0
pf00270	(DEAD)	0	0	0	1
pf00383	(dCMP_cyt_deam_1)	0	1	0	1
pf00817	(IMS)	0	9	0	5
pf01145	(Band_7)	0	3	0	1
pf01343	(Peptidase_S49)	1	0	1	0
pf01368	(DHH)	0	0	1	1
pf01522	**(Polysacc_deac_1)**	0	1	0	2
pf03838	(RecU)	1	6	2	6
pf05954	(Phage_GPD)	3	3	0	2
pf07833	(Cu_amine_oxidN1)	0	5	0	8
pf11798	(IMS_HHH)	0	6	0	5
pf00180	(Iso_dh)	0	0	1	0
pf00246	(Peptidase_M14)	0	2	0	7
pf00462	(Glutaredoxin)	0	3	0	1
pf00580	(UvrD-helicase)	0	2	1	2
pf02384	(N6_Mtase)	0	0	0	3
pf02511	(Thy1)	0	2	1	6
pf02954	(HTH_8)	1	0	0	0
pf03869	(Arc)	5	3	3	6
pf04448	(DUF551)	3	2	1	5
pf05565	(Sipho_Gp157)	2	4	3	6
pf05876	(Terminase_GpA)	1	2	5	3
pf06763	(Minor_tail_Z)	3	2	3	2
pf06805	(Lambda_tail_I)	3	2	4	4
pf06946	(Phage_holin_5_1)	1	1	0	3
pf09355	(Phage_Gp19)	0	1	0	5
pf09718	(Tape_meas_lam_C)	3	3	6	6
pf10765	(DUF2591)	6	5	1	5
pf11133	(Phage_head_fibr)	0	2	1	7
pf11417	(Inhibitor_G39P)	0	1	0	3
pf11611	(DUF4352)	0	2	1	4
pf12836	(HHH_3)	0	1	0	3
pf13227	(DUF4035)	3	2	3	4
pf13472	(Lipase_GDSL_2)	0	2	0	1
pf13495	(Phage_int_SAM_4)	0	7	3	4
pf14398	(ATPgrasp_YheCD)	0	1	1	7
pf15943	**(YdaS_antitoxin)**	3	1	3	4
pf17318	(DUF5361)	0	1	1	5
pf00011	(HSP20)	1	0	0	0
pf01757	(Acyl_transf_3)	3	5	1	7
pf01773	(Nucleos_tra2_N)	0	0	0	0
pf01966	(HD)	0	1	1	1
pf02413	(Caudo_TAP)	3	4	7	3
pf02811	(PHP)	0	3	0	1
pf04984	(Phage_sheath_1)	2	5	0	3
pf05343	(Peptidase_M42)	0	0	1	0

pf08343	(RNR_N)	0	3	2	3
pf08460	(SH3_5)	3	7	2	4
pf09466	(Yqai)	0	2	2	5
pf10076	(DUF2313)	1	5	1	4
pf10711	(DUF2513)	0	4	0	4
pf13614	(AAA_31)	2	2	1	2
pf14284	(PcfJ)	0	2	1	4
pf14594	(Sipho_Gp37)	0	2	2	7
pf00023	(Ank)	4	0	0	0
pf00472	(RF-1)	0	1	0	1
pf01507	(PAPS_reduct)	2	1	1	3
pf01661	(Macro)	0	1	3	4
pf02272	(DHHA1)	0	0	1	0
pf03589	(Antiterm)	2	2	3	3
pf04266	(ASCH)	3	2	1	3
pf04932	(Wzy_C)	0	2	0	5
pf04965	(GPW_gp25)	1	1	0	0
pf04985	(Phage_tube)	3	2	0	1
pf05136	(Phage_portal_2)	1	2	3	2
pf05272	**(VirE)**	0	0	0	3
pf05489	(Phage_tail_X)	2	3	0	2
pf05869	(Dam)	7	7	2	9
pf06183	(DinI)	3	1	0	2
pf06222	(Phage_TAC_1)	3	2	3	3
pf06289	(FlbD)	0	2	0	4
pf06531	(DUF1108)	1	1	0	3
pf07902	(Gp58)	0	6	2	4
pf08707	(PriCT_2)	0	3	0	4
pf09684	(Tail_P2_I)	2	3	0	2
pf09693	(Phage_XkdX)	1	4	2	7
pf11672	(DUF3268)	0	1	1	4
pf13518	(HTH_28)	0	0	0	1
pf13604	(AAA_30)	0	3	1	2
pf13646	(HEAT_2)	0	1	0	0
pf13708	(DUF4942)	0	3	1	3
pf14471	(DUF4428)	0	0	0	4
pf16083	(Phage_holin_3_3)	1	1	0	2
pf16473	(DUF5051)	3	2	3	3
pf16931	(Phage_holin_8)	3	3	2	3
pf17356	(PBSX_XtrA)	0	5	1	6
pf17482	(Phage_sheath_1C)	2	4	1	3
pf00078	(RVT_1)	0	2	0	2
pf00226	(DnaJ)	0	1	0	1
pf01068	(DNA_ligase_A_M)	0	6	2	4
pf02195	(ParBc)	0	5	1	2
pf02768	(DNA_pol3_beta_3)	0	0	0	3
pf02810	(SEC-C)	0	1	0	0
pf04011	(LemA)	0	0	1	1
pf05036	(SPOR)	0	3	0	1
pf05930	(Phage_AlpA)	1	3	0	2
pf06992	(Phage_lambda_P)	3	3	2	4
pf07972	(Flavodoxin_NdrI)	0	3	1	3
pf09979	(DUF2213)	4	4	3	4
pf10934	(DUF2634)	0	4	0	4

pf11726	(Inovirus_Gp2)	0	0	0	1
pf11863	(DUF3383)	2	3	2	1
pf13238	(AAA_18)	0	2	1	2
pf13545	(HTH_Crp_2)	0	1	0	1
pf13671	(AAA_33)	0	1	1	1
pf16080	(Phage_holin_2_3)	2	2	1	2
pf16784	(HNHc_6)	3	6	1	4
pf00075	(RNase_H)	0	0	1	0
pf00533	(BRCT)	1	1	0	0
pf02599	(CsrA)	0	1	0	1
pf04002	(RadC)	1	0	0	0
pf04095	(NAPRTase)	0	0	1	0
pf04101	(Glyco_tran_28_C)	0	0	0	0
pf04245	(NA37)	0	1	0	1
pf04381	(RdgC)	0	1	0	2
pf04480	(DUF559)	1	1	0	2
pf04492	(Phage_rep_O)	2	5	2	8
pf04717	(Phage_base_V)	2	2	0	1
pf05014	(Nuc_deoxyrib_tr)	0	1	1	3
pf05106	(Phage_holin_3_1)	3	1	4	1
pf05263	(DUF722)	0	0	1	2
pf05766	(NinG)	4	1	3	1
pf05926	(Phage_GPL)	3	2	1	1
pf06141	(Phage_tail_U)	2	1	3	1
pf06147	(DUF968)	2	1	0	2
pf06254	**(YdaT_toxin)**	2	3	2	2
pf06995	(Phage_P2_GpU)	2	3	0	2
pf07498	(Rho_N)	0	0	0	3
pf07501	(G5)	0	0	2	3
pf07532	**(Big_4)**	0	2	1	3
pf07751	**(Abi_2)**	0	1	0	3
pf07761	(DUF1617)	1	1	0	2
pf07768	**(PVL_ORF50)**	1	3	2	4
pf07825	(Exc)	0	0	1	1
pf08765	(Mor)	0	2	2	3
pf09003	(Arm-DNA-bind_1)	2	1	1	3
pf09327	(DUF1983)	2	1	4	3
pf09639	(YjcQ)	0	0	0	2
pf10263	(SprT-like)	0	0	0	1
pf10782	(zf-C2HCIx2C)	0	5	2	3
pf10960	(Holin_BhlA)	0	2	2	2
pf12200	(DUF3597)	0	1	1	4
pf12236	(Head-tail_con)	1	3	0	3
pf13175	(AAA_15)	0	3	1	5
pf13229	(Beta_helix)	0	2	1	2
pf13304	(AAA_21)	1	0	1	3
pf13365	(Trypsin_2)	0	1	1	1
pf13505	(OMP_b-brl)	0	0	0	1
pf13538	(UvrD_C_2)	0	3	1	1
pf13589	(HATPase_c_3)	1	0	0	0
pf13637	(Ank_4)	3	0	0	0
pf13856	(Gifsy-2)	1	2	2	2
pf14549	(P22_Cro)	0	1	0	2
pf16729	(DUF5067)	0	1	0	4

pf00186	(DHFR_1)	0	3	1	2
pf01541	(GIY-YIG)	0	2	0	1
pf01653	(DNA_ligase_aden)	0	0	0	1
pf04327	(Peptidase_Prp)	0	3	1	2
pf04355	(SmpA_OmlA)	0	1	1	1
pf04383	(KilA-N)	4	1	2	1
pf05708	(Peptidase_C92)	0	0	0	1
pf06146	(PsiE)	1	1	1	1
pf06440	(DNA_pol3_theta)	2	3	0	6
pf07105	(DUF1367)	2	3	0	1
pf07852	(DUF1642)	1	6	2	1
pf10417	**(1-cysPrx_C)**	0	0	0	1
pf13192	(Thioredoxin_3)	0	1	1	1
pf13649	(Methyltransf_25)	0	1	0	0
pf16786	(RecA_dep_nuc)	2	3	0	2
pf00395	(SLH)	0	2	0	2
pf00493	(MCM)	0	0	0	2
pf01119	(DNA_mis_repair)	1	0	0	0
pf01258	(zf-dskA_traR)	1	1	1	1
pf01527	(HTH_Tnp_1)	0	1	0	1
pf01726	(LexA_DNA_bind)	0	1	2	4
pf02316	(HTH_Tnp_Mu_1)	0	1	1	1
pf02452	**(PemK_toxin)**	0	1	1	1
pf02547	(Queuosine_synth)	0	0	0	1
pf02556	(SecB)	0	1	0	0
pf02655	(ATP-grasp_3)	0	1	0	1
pf02732	(ERCC4)	0	0	1	1
pf02796	(HTH_7)	1	1	1	2
pf03887	(YfbU)	0	0	1	1
pf04336	**(ACP_PD)**	0	0	0	1
pf04952	(AstE_AspA)	0	0	1	1
pf05125	(Phage_cap_P2)	1	1	0	0
pf05449	(Phage_holin_3_7)	1	2	1	1
pf05534	(HicB)	0	0	0	1
pf05772	(NinB)	6	0	4	4
pf05895	(DUF859)	0	0	0	2
pf05944	(Phage_term_smal)	1	1	0	0
pf06120	(Phage_HK97_TLTM)	5	5	5	8
pf06223	(Phage_tail_T)	1	1	3	1
pf06252	(DUF1018)	0	1	1	2
pf06322	(Phage_NinH)	3	0	2	2
pf06576	(DUF1133)	2	1	2	1
pf06689	(zf-C4_ClpX)	0	1	1	3
pf06791	(TMP_2)	0	1	3	1
pf06855	(YozE_SAM_like)	0	0	1	2
pf06891	(P2_Phage_GpR)	2	1	0	0
pf06894	(Phage_TAC_2)	1	1	4	1
pf06908	(DUF1273)	0	0	1	0
pf07129	(DUF1381)	1	2	2	3
pf07453	(NUMOD1)	0	0	0	2
pf07553	(Lipoprotein_Ltp)	0	0	0	1
pf07733	(DNA_pol3_alpha)	0	3	0	2
pf08291	(Peptidase_M15_3)	1	1	0	1
pf08676	(MutL_C)	1	0	0	0

pf08755	(YccV-like)	0	0	0	0
pf08813	(Phage_tail_3)	2	3	2	2
pf08867	(FRG)	0	1	1	1
pf08875	(DUF1833)	4	1	3	3
pf09299	(Mu-transpos_C)	0	1	1	2
pf09956	(DUF2190)	0	1	2	1
pf09992	(NAGPA)	0	1	0	2
pf10109	(Phage_TAC_7)	2	1	0	0
pf10124	(Mu-like_gpT)	0	1	1	0
pf10127	(Nuc-transf)	0	3	1	1
pf10269	(Tmemb_185A)	0	2	2	2
pf10543	(ORF6N)	2	4	3	4
pf10656	(DUF2483)	1	1	1	3
pf10781	(DSRB)	0	0	1	0
pf10809	(DUF2732)	1	0	0	0
pf10844	(DUF2577)	0	2	0	2
pf10926	(DUF2800)	0	1	0	1
pf10979	(DUF2786)	0	1	0	2
pf10991	(DUF2815)	0	1	0	1
pf11023	(DUF2614)	0	0	0	1
pf11213	(DUF3006)	0	1	0	3
pf11236	(DUF3037)	1	0	0	2
pf11300	(DUF3102)	1	3	0	3
pf11363	(DUF3164)	0	1	1	2
pf11443	(DUF2828)	0	2	0	2
pf11588	(DUF3243)	0	1	0	1
pf12167	(Arm-DNA-bind_2)	2	2	2	1
pf12705	(PDDEXK_1)	1	3	1	1
pf12789	(PTR)	0	1	0	1
pf12949	(HeH)	0	1	1	2
pf13148	(DUF3987)	0	0	0	1
pf13306	**(LRR_5)**	0	1	1	2
pf13401	(AAA_22)	0	1	1	2
pf13455	(MUG113)	0	0	0	1
pf13599	(Pentapeptide_4)	0	1	0	1
pf13638	**(PIN_4)**	0	1	1	1
pf13643	(DUF4145)	0	1	0	1
pf13693	(HTH_35)	0	1	1	1
pf13973	(DUF4222)	1	0	1	1
pf14058	(PcfK)	0	0	0	1
pf14213	(DUF4325)	0	2	1	2
pf14279	(HNH_5)	0	2	0	2
pf14490	(HHH_4)	0	3	1	1
pf14528	(LAGLIDADG_3)	0	3	2	3
pf14890	(Intein_splicing)	0	2	0	2
pf16227	(DUF4886)	0	3	1	2
pf16461	(Phage_TTP_12)	2	1	3	1
pf16462	(Phage_TAC_14)	1	0	0	1
pf16463	(Phage_TTP_13)	1	0	0	1
pf17236	(DUF5309)	0	3	0	2
pf17282	(DUF5347)	1	0	0	0
pf17443	(pXO2-72)	0	1	0	0
pf00531	**(Death)**	0	1	0	1
pf00596	(Aldolase_II)	0	0	0	0

pf00625	(Guanylate_kin)	0	1	1	1
pf01385	(OrfB_IS605)	0	2	0	3
pf01402	(RHH_1)	0	1	0	0
pf01734	(Patatin)	1	0	0	0
pf06381	(DUF1073)	6	6	5	5
pf07282	(OrfB_Zn_ribbon)	0	2	0	3
pf08085	**(Entericidin)**	1	1	1	0
pf08719	(DUF1768)	0	0	1	0
pf09950	(DUF2184)	2	3	2	1
pf10122	(Mu-like_Com)	0	1	0	2
pf11041	(DUF2612)	2	3	2	2
pf12571	(DUF3751)	1	2	0	0
pf13262	(DUF4054)	2	3	2	1
pf14072	(DndB)	0	0	1	1
pf00041	(fn3)	0	2	0	1
pf00136	(DNA_pol_B)	0	1	0	1
pf00227	(Proteasome)	0	0	0	1
pf00415	(RCC1)	0	2	0	1
pf00804	(Syntaxin)	0	1	0	1
pf01183	(Glyco_hydro_25)	0	1	1	3
pf01503	(PRA-PH)	0	2	0	1
pf01850	**(PIN)**	0	1	0	1
pf01896	(DNA_primase_S)	0	0	0	1
pf01935	(DUF87)	0	0	0	1
pf01943	(Polysacc_synt)	0	0	0	1
pf02005	(TRM)	0	0	1	1
pf02018	**(CBM_4_9)**	0	1	0	1
pf02037	(SAP)	0	1	0	1
pf02735	(Ku)	0	2	0	1
pf02831	(gpW)	1	0	1	0
pf02924	(HDPD)	1	0	1	0
pf03079	(ARD)	0	0	0	1
pf03217	(SLAP)	0	1	1	1
pf03235	(DUF262)	1	3	1	1
pf03406	(Phage_fiber_2)	1	0	0	0
pf04014	**(MazE_antitoxin)**	0	0	0	1
pf04070	(DUF378)	0	1	0	1
pf04297	(UPF0122)	0	0	0	1
pf04550	(Phage_holin_3_2)	1	0	0	0
pf04570	(zf-FLZ)	0	0	0	1
pf04606	(Ogr_Delta)	1	1	0	0
pf04971	(Phage_holin_2_1)	0	0	1	0
pf05063	(MT-A70)	2	1	1	3
pf05069	(Phage_tail_S)	1	2	0	0
pf05180	(zf-DNL)	0	0	0	1
pf05183	(RdRP)	0	2	0	1
pf05339	(DUF739)	0	1	0	1
pf05382	**(Amidase_5)**	0	1	0	1
pf05478	(Prominin)	0	1	0	1
pf05635	(23S_rRNA_IVP)	0	1	0	1
pf05658	**(YadA_head)**	1	1	1	1
pf05662	**(YadA_stalk)**	1	1	1	1
pf05840	(Phage_GPA)	1	0	0	0
pf05901	(Excalibur)	0	0	0	1

pf05929	(Phage_GPO)	1	1	0	0
pf06048	(DUF927)	1	1	1	1
pf06054	(CoiA)	0	1	0	1
pf06056	(Terminase_5)	1	1	0	0
pf06074	(DUF935)	0	1	1	0
pf06116	(RinB)	1	0	1	3
pf06291	**(Lambda_Bor)**	1	0	1	0
pf06301	**(Lambda_Kil)**	1	0	1	1
pf06323	(Phage_antiter_Q)	0	1	0	1
pf06406	(StbA)	0	1	0	1
pf06519	(TolA)	0	0	0	1
pf06688	(DUF1187)	0	0	1	1
pf06890	(Phage_Mu_Gp45)	1	1	1	1
pf06892	(Phage_CP76)	1	0	0	0
pf06906	(DUF1272)	0	0	0	1
pf07022	(Phage_CI_repr)	1	0	0	0
pf07030	(DUF1320)	0	1	0	0
pf07041	(DUF1327)	0	0	1	0
pf07102	(DUF1364)	1	0	1	0
pf07157	(DNA_circ_N)	0	1	1	1
pf07166	(DUF1398)	1	0	1	0
pf07274	(DUF1440)	1	0	0	0
pf07278	(DUF1441)	0	1	0	1
pf07409	(GP46)	1	1	1	1
pf07581	**(Glug)**	0	1	0	1
pf07866	(DUF1653)	0	1	0	0
pf07874	(DUF1660)	0	0	0	1
pf07878	(RHH_5)	0	0	1	1
pf08273	(Prim_Zn_Ribbon)	1	1	1	2
pf08275	(Toprim_N)	0	1	1	0
pf08822	(DUF1804)	0	1	1	0
pf08861	(DUF1828)	1	0	0	0
pf08862	(DUF1829)	1	0	0	0
pf08890	(Phage_TAC_5)	0	2	0	1
pf08963	(DUF1878)	1	0	0	0
pf09008	(Head_binding)	2	0	0	1
pf09048	(Cro)	5	2	3	3
pf09369	(DUF1998)	0	1	1	1
pf09372	(PRANC)	1	0	0	0
pf09393	(DUF2001)	0	2	0	1
pf09424	(YqeY)	0	0	1	0
pf09642	(YonK)	0	2	0	1
pf09682	(Phage_holin_6_1)	0	3	1	1
pf09953	(DUF2187)	0	1	0	0
pf10108	(DNA_pol_B_exo2)	1	1	0	1
pf10123	(Mu-like_Pro)	0	1	0	0
pf10544	(T5orf172)	3	2	3	4
pf10546	(P63C)	0	0	1	0
pf10548	(P22_AR_C)	0	0	1	0
pf10549	(ORF11CD3)	1	0	0	0
pf10668	(Phage_terminase)	0	3	0	0
pf10758	(DUF2586)	1	0	0	0
pf10761	(DUF2590)	1	0	0	0
pf10772	(DUF2597)	1	0	0	0

pf10800	(DUF2528)	1	0	1	1
pf10828	(DUF2570)	1	1	1	1
pf10834	(DUF2560)	2	2	1	2
pf10883	(DUF2681)	1	0	0	0
pf10963	(Phage_TAC_10)	1	0	0	0
pf11008	(DUF2846)	0	0	1	0
pf11058	(Ral)	1	0	1	1
pf11166	(DUF2951)	0	0	0	1
pf11192	(DUF2977)	1	3	1	3
pf11195	(DUF2829)	0	5	1	1
pf11225	(DUF3024)	1	0	0	1
pf11423	(Repressor_Mnt)	3	1	1	2
pf11438	(N36)	1	0	1	0
pf11494	(Ta0938)	0	0	0	1
pf11553	(DUF3231)	0	2	0	0
pf11673	(DUF3269)	0	1	0	1
pf11681	(DUF3277)	0	1	0	0
pf11839	(Alanine_zipper)	1	0	0	0
pf12227	(DUF3603)	0	0	1	1
pf12358	(DUF3644)	0	1	0	0
pf12651	(RHH_3)	0	0	1	0
pf12686	(DUF3800)	0	1	0	1
pf12708	(Pectate_lyase_3)	0	3	1	3
pf12773	(DZR)	0	1	0	1
pf12784	(PDDEXK_2)	1	0	0	0
pf12810	(Gly_rich)	0	1	0	1
pf12869	(tRNA_anti-like)	0	1	1	1
pf12961	(DUF3850)	0	2	0	0
pf13205	**(Big_5)**	0	1	0	1
pf13245	(AAA_19)	0	2	0	1
pf13264	(DUF4055)	0	0	0	1
pf13269	(DUF4060)	0	0	0	2
pf13338	**(AbiEi_4)**	1	0	0	0
pf13362	(Toprim_3)	1	1	1	2
pf13374	(TPR_10)	1	0	0	0
pf13384	(HTH_23)	0	1	0	1
pf13403	(Hint_2)	0	0	0	1
pf13424	(TPR_12)	1	0	0	0
pf13683	(rve_3)	0	0	0	1
pf13702	(Lysozyme_like)	0	1	0	1
pf13789	(DUF4181)	0	0	0	2
pf13857	(Ank_5)	1	0	0	0
pf13936	(HTH_38)	0	0	0	1
pf13974	(YebO)	1	1	1	1
pf13986	(DUF4224)	1	1	1	1
pf14022	(DUF4238)	0	1	0	1
pf14163	(SieB)	1	0	1	1
pf14253	**(AbiH)**	0	0	0	1
pf14297	(DUF4373)	0	0	0	1
pf14305	(ATPgrasp_TupA)	0	0	1	0
pf14354	(Lar_restr_allev)	3	1	3	2
pf14359	(DUF4406)	1	1	1	1
pf14397	(ATPgrasp_ST)	1	0	0	1
pf14470	(bPH_3)	0	1	0	0

pf14472	(DUF4429)	0	1	0	1
pf14486	(DUF4432)	0	0	0	0
pf14489	(QueF)	0	0	0	0
pf14493	(HTH_40)	0	1	1	1
pf14667	(Polysacc_synt_C)	0	0	0	1
pf14743	(DNA_ligase_OB_2)	0	1	1	0
pf14897	(EpsG)	0	1	1	0
pf15944	(DUF4752)	1	0	1	1
pf16075	(DUF4815)	1	0	0	0
pf16452	(Phage_CI_C)	1	0	0	0
pf16459	(Phage_TAC_13)	1	1	0	0
pf16460	(Phage_TTP_11)	1	1	0	0
pf16807	(DUF5072)	0	1	0	1
pf17212	(Tube)	1	2	0	1
pf17363	(DUF5388)	1	0	0	0
pf17399	(DUF5405)	0	0	0	1
pf00910	(RNA_helicase)	1	0	0	0
pf01051	(Rep_3)	0	1	0	0
pf01712	(dNK)	0	0	0	1
pf02407	(Viral_Rep)	1	0	0	0
pf03009	(GDPD)	0	1	0	0
pf03193	(RsgA_GTPase)	0	1	0	1
pf04679	(DNA_ligase_A_C)	0	2	0	1
pf08212	**(Lipocalin_2)**	1	1	1	0
pf08305	(NPCBM)	0	0	0	1
pf08937	(DUF1863)	0	1	0	1
pf12102	(DUF3578)	0	1	0	1
pf12323	(HTH_OrfB_IS605)	0	0	0	1
pf14015	(DUF4231)	0	1	0	1
pf14579	(HHH_6)	0	1	0	1
pf15975	**(Flot)**	0	1	0	1
pf00249	(Myb_DNA-binding)	0	0	1	0
pf00688	(TGFb_propeptide)	0	1	0	0
pf00754	(F5_F8_type_C)	0	1	0	0
pf01978	(TrmB)	0	1	0	0
pf02061	**(Lambda_CIII)**	0	0	1	0
pf02586	(SRAP)	0	1	0	0
pf02661	(Fic)	0	1	0	0
pf03335	(Phage_fiber)	0	1	1	1
pf04255	(DUF433)	0	1	0	0
pf04313	(HSDR_N)	0	1	0	0
pf04375	(HemX)	0	1	0	0
pf04471	(Mrr_cat)	0	2	0	0
pf04870	(Moulting_cycle)	0	0	0	1
pf05067	(Mn_catalase)	0	1	0	0
pf05269	(Phage_CII)	4	1	2	2
pf05354	(Phage_attach)	0	0	1	0
pf06042	(NTP_transf_6)	0	1	0	0
pf06064	(Gam)	0	0	1	0
pf06085	(Rz1)	1	0	0	0
pf06260	(DUF1024)	0	0	1	1
pf06296	**(RelE)**	0	1	0	0
pf06316	**(Ail_Lom)**	0	0	2	0
pf06600	(DUF1140)	0	0	0	1

pf06941	(NT5C)	0	1	0	0
pf07026	(DUF1317)	1	0	1	0
pf07131	(DUF1382)	1	0	1	1
pf07471	(Phage_Nu1)	0	0	1	0
pf07484	(Collar)	0	0	0	1
pf07659	(DUF1599)	1	1	0	0
pf07691	**(PA14)**	0	1	0	0
pf07693	(KAP_NTPase)	1	0	0	0
pf08357	**(SEFIR)**	0	0	0	1
pf08868	(YugN)	0	1	0	1
pf08873	(DUF1834)	0	1	0	0
pf09306	(Phage-scaffold)	1	1	0	1
pf09537	(DUF2383)	0	1	0	0
pf10065	(DUF2303)	0	1	0	0
pf10088	(DUF2326)	0	0	0	1
pf10547	(P22_AR_N)	1	1	1	1
pf10743	(Phage_Cox)	0	1	0	0
pf10881	(DUF2726)	0	1	0	0
pf11039	(DUF2824)	1	1	0	1
pf11134	(Phage_stabilise)	1	1	0	1
pf11436	(DUF3199)	0	1	0	0
pf11650	(P22_Tail-4)	0	1	0	1
pf11651	(P22_CoatProtein)	1	1	0	1
pf11750	(DUF3307)	0	1	0	0
pf11962	(Peptidase_G2)	0	0	1	2
pf12206	(DUF3599)	0	1	0	0
pf12421	(DUF3672)	0	0	3	0
pf12677	(DUF3797)	0	1	0	0
pf12873	(DUF3825)	0	1	0	0
pf13088	**(BNR_2)**	0	1	0	0
pf13451	(zf-trcl)	0	1	0	0
pf13540	(RCC1_2)	0	1	0	0
pf13550	(Phage-tail_3)	2	1	4	2
pf13619	(KTSC)	0	1	0	0
pf13876	(Phage_gp49_66)	1	1	0	1
pf13895	**(Ig_2)**	1	1	1	1
pf13935	(Ead_Ea22)	1	0	1	1
pf14000	(Packaging_FI)	0	0	1	0
pf14216	(DUF4326)	0	1	0	0
pf14264	(Glucos_trans_II)	0	1	0	0
pf14311	(DUF4379)	0	1	0	0
pf14367	(DUF4411)	0	1	0	0
pf14386	(DUF4417)	0	2	0	0
pf14436	**(EndoU_bacteria)**	0	1	0	0
pf14550	(Peptidase_S78_2)	0	1	0	0
pf15525	(DUF4652)	0	1	0	0
pf15968	(RexB)	1	0	2	0
pf15969	(RexA)	1	0	2	0
pf16082	(Phage_holin_2_4)	0	2	0	0
pf16162	(DUF4868)	0	1	0	0
pf16308	(DUF4950)	0	1	0	0
pf16510	(P22_portal)	1	1	0	1
pf16928	(Inj_translocase)	1	1	0	1
pf16938	(Phage_holin_Dp1)	1	5	0	1

pf17400	(DUF5406)	0	1	0	0
pf17466	(NinD)	0	0	2	0
pf17481	(Phage_sheath_1N)	0	1	0	0
