# Supplementary material for: Finer-Scale Phylosymbiosis: Insights from Insect Viromes
Source: mSystems. 2018 Dec 18;3(6):e00131-18. doi: 10.1128/mSystems.00131-18 (PMC6299154; doi:10.1128/mSystems.00131-18)
Supplement: FIG S1 [file sys006182303sf1.pdf]

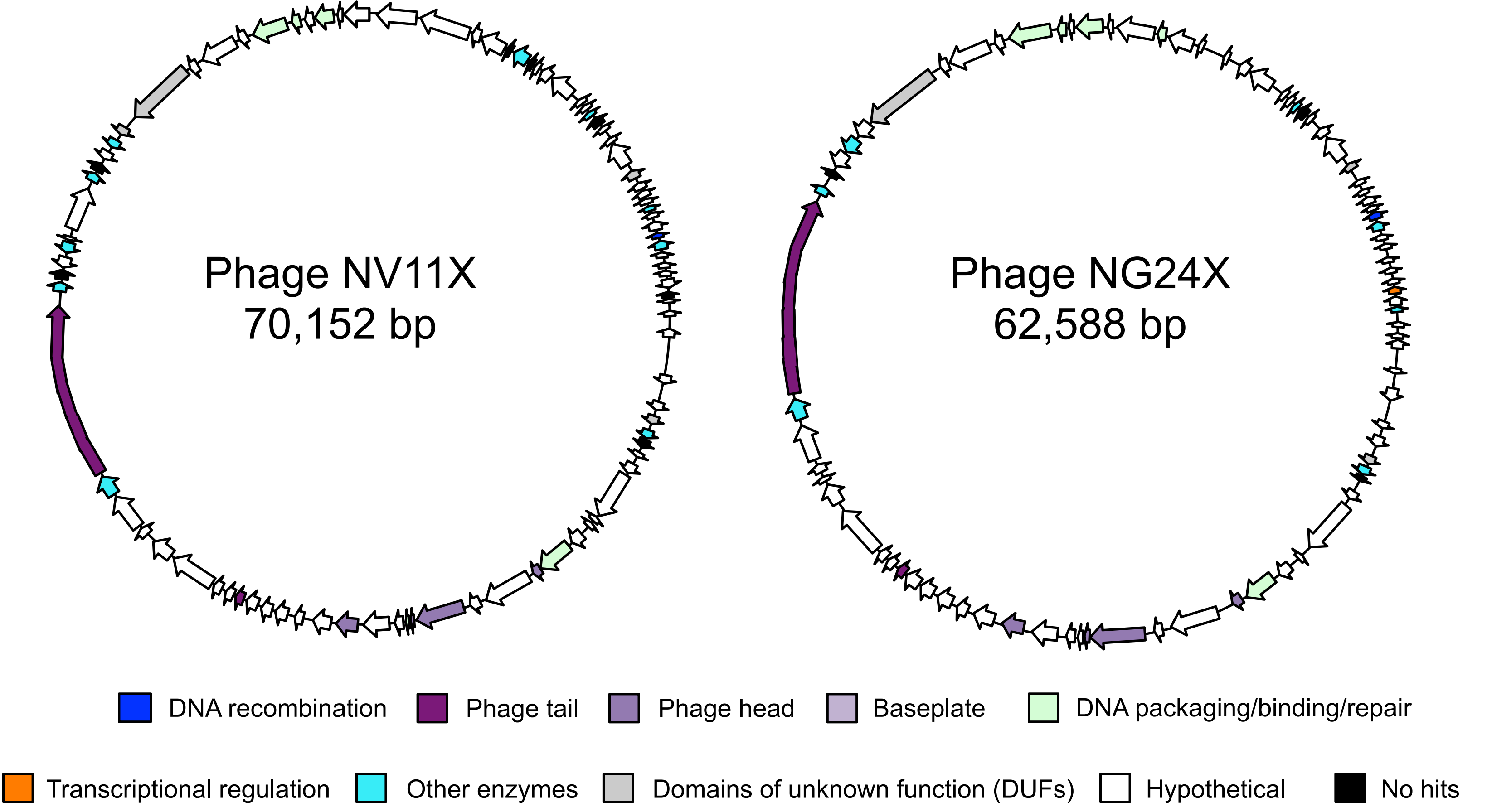
Phage NV11X
70,152 bp
Phage NG24X
62,588 bp
DNA recombination
Phage tail
Phage head
Baseplate
DNA packaging/binding/repair
Transcriptional regulation
Other enzymes
Domains of unknown function (DUFs)
Hypothetical
No hits
